# Using the Lens Database for Staff Publications

**DOI:** 10.5195/jmla.2020.918

**Published:** 2020-04-01

**Authors:** Rob Penfold

**Affiliations:** Director, Library and Literacy, Barwon Health, Geelong, VIC, Australia, rob.penfold@barwonhealth.org.au, https://orcid.org/0000-0002-5879-7388

## INTRODUCTION

In a rapidly changing landscape, many health sciences libraries are looking to increase the support they provide for research in their organization [1–3]. The Lens database is a free platform for enabling discovery of both scholarly and patent literature. Started in 1998 as Patent Lens, its initial focus was to provide more transparent access to the patent literature. The Lens About page states that within two years, they expect to host over 95% of the world’s patent literature. More recently it has also started merging scholarly literature into the database [4], sourced from several providers but primarily from PubMed, CrossRef, and Microsoft Academic Graph.

With more than 200 million scholarly works, it is one of the largest indexes currently available [5], and this combination of scholarly and patent information provides a powerful means for investigating linkages between research and innovation [6]. The Lens is a collaboration between the not-for-profit Cambia and Queensland University of Technology. It has received funding from the Rockefeller Foundation and the Bill and Melinda Gates Foundation, amongst others. It is noncommercial in nature and aims to facilitate innovation by providing free tools that help highlight linkages between the research and commercial literatures.

## USE CASE FOR STAFF PUBLICATIONS

It is beyond the scope of this short review to cover all the features provided by the Lens. Instead, it will focus on the use case of providing access to, and highlighting, an organization’s research output, given that the Lens provides a number of appealing options in this regard. It is free, which makes it appealing for health services libraries that often have limited budgets, compared to universities and research institutes. It offers one of the largest citation indexes available, which means coverage of an institution’s output should be reasonably complete [5].

While it is possible to search by researcher affiliation, it is worth noting that the Lens sources many of their citations from Microsoft Academic Graph and that this resource may have missing or wrong affiliation information, compared to commercial indexes such as Web of Science or Scopus [7]. However, this is not a barrier to the proposed use case because records can both be imported into the Lens and added to a collection on an individual basis in the platform. The Lens allows linkage to a library’s collection via the WorldCat Registry, which is free to use for all libraries.

Citing patents and citing articles can be viewed. A number of research metric visualizations are available, including most-frequent authors, disciplines, journals, funding sources, and institutions, with extensive custom visualizations also available. There is an application programming interface (API) available that can be used, for example, to automatically display a list of an organization’s most recent staff publications on its research page.

## PROCESS

A six-step process is used for both creating a staff publications collection in the Lens and then highlighting this research output. For simplicity, only journal articles have been utilized in the example.

1. Create an account. A free Lens account can be created; alternatively ORCID or LinkedIn credentials can be used.
2. Create a set of staff publications. The Lens provides two options for affiliation search. Firstly, the Institution filter in the left-hand panel can be applied. Alternatively, the Edit Search option, which is effectively the advanced search option, allows searching in an Author affiliation field, and this approach seems to produce more results than the first one. For the reviewer’s institution (Barwon Health), the number of staff publications that the Lens identified was substantially fewer than the number identified by searching several databases independently. However, the Lens allows the importation of external records, so it is possible to identify staff publications separately and simply use the Lens as a holding platform for these records. This can be achieved by creating a Collection in the Work Area and then importing a set of identifications (IDs), such as digital object identifier (DOI), PubMed ID (PMID), and more. The set should appear almost instantly; if not, the incorrect format of the ID may have been used. For publications without such an ID, records can be identified in the Lens by search and selection, and then added to the collection.
3. Enable access to the library collection. The Lens provides links to some full text natively via the “Open Access” or “Full Text” links in individual records. Additionally, every scholarly record has a “Find full-text at your institution” link, which can be connected with the WorldCat Registry [8]. Libraries that use OCLC products would generally already have an entry in this registry. The WorldCat Registry is free for all libraries (and several other types of institutions), so an entry can be created to facilitate access to a library’s collection. This can be achieved by including the link resolver in the OpenURL Resolvers section, and the Internet protocol (IP) range (including any proxy IP for offsite access) in the IP Addresses section. As an aside, the combination of linkage to a library’s collection and a large index with many search options means that the Lens platform could potentially function as a form of a free discovery layer.
4. Set Collection access level. Once a collection of staff publications has been created, then the access level can be set. The default is Restricted (only account holder can view), but this can be changed to either Limited (anyone with the link can view) or Public (discoverable by search). As an example, the 2019 journal article research output for Barwon Health can be viewed at Barwon Health Research Output - 2019. If the sort option for a Collection is changed, then the resulting uniform resource locator (URL) is persistent. In this way, it would be possible to create links that, for instance, display the most recent publications first or show staff publications that have been cited the most.
5. Use research metric visualizations. By default, a collection will display several visualizations related to that collection, such as the most frequent institutions, publications over time, geographic information, authors, sources, and subjects. Via the Analysis tab, it is possible to delete, edit, and add new visualizations, and the order of visualizations can be changed by dragging them up or down. This visualization configuration can be saved as an Analysis Dashboard and is applied to that set of search results. These visualizations could be utilized in three potential ways. Firstly, by directly linking to the analysis dashboard from relevant organizational pages. Several research visualizations relating to Barwon Health publications in 2019 can be viewed in a second sample Barwon Health Research Output - 2019. Secondly, to provide a more immediate visual impact, visualizations can be downloaded as image files, uploaded to the research site, and updated as required. Finally, it is possible to place the dashboard URL inside an iframe on the research site to provide embedded live visuals. Research metrics visualizations have been used to support researcher grant applications, so this could be an additional research support service that the library provides.
6. Create researcher profiles (optional extra). These are common for large research organizations. A version of these can be created from the staff publications collection, which can be achieved by using the Authors filter on the left. A permalink to the resulting set can be obtained by using “Share Results” to obtain a shortened URL. Alternatively, the “Save Query” option allows the set to be given a title and description (which can include links), as well as to elect to receive email notifications for any new records that appear in the set. A number of sort options are available (e.g., date, times cited), and links for such sorted sets are persistent. In this manner, links can be created that highlight the output and influence (via scholarly and patent citations) of individual researchers and can be used on the research site. The Lens also provides subject limits. Using these would not match departmental research output exactly but could provide useful entry points to organizational research around a given topic [9].

## ADDITIONAL USEFUL FEATURES

The process outlined above will provide an extended picture of an institution’s research output compared to other approaches, such as creating a set of staff publications in PubMed or creating publication repositories using platforms such as DSpace. However, further extension of the research position is possible due to a number of additional tools in the Lens. Given their nature, their usefulness is probably more applicable to organizations that produce a substantial research output, such as research institutes, universities, or large health services. PatCite is a tool that can be used to provide insight into the relationship between scholarly works and the patents that cite them [10]. PatSeq is itself a collection of five tools that allows the searching and analysis of biological sequences (DNA, RNA, protein) in patents, including some sequences that are only disclosed in the patent literature [11]. It is worth noting that all mapped patent sequences relating to the human genome are available here. The In4M tool uses citation-based metrics to map the influence of research on industry and innovation, potentially facilitating collaborations and funding partnerships [12]. It currently covers universities and large research institutes only but will be significantly expanded in 2020 [13].

Another valuable aspect of the Lens is the ability to retrieve both citing patents and articles. For an individual record, these can be viewed via the Relevant tab. If these are then viewed in Patent or Scholar Search (via the green box that appears), it is possible to set up an email alert for new citing works via the “Save Query” option. The situation is different for a collection. Citing patents works in the same way, and an alert can be similarly enabled. Alerting researchers to such citing patents could be a valuable service in terms of seeing how their research is being applied and could potentially inform new collaborations and directions. Articles citing a collection are not viewable as a set; only a total number is currently available via the Scholarly Citations tab. However, the Lens staff are looking to further develop capabilities in this area. It is possible to view the set of articles in the collection that have themselves been cited (“Works cited by Scholarly”). Setting up an alert as above for this set would allow identification of staff publications that have been cited for the first time and perhaps could be used as the basis for a congratulatory email to the researchers involved.

Research metrics are not just limited to organizations. By default, the Lens displays all scholarly works, so it is very easy to use the Country filter on the left to limit to a particular country’s research output. This could be used to identify the most active institutions, authors, funding sources, collaborating countries, and more, while flag limits such “Cited by Patents” and “Cited by Scholarly” can be applied to highlight impactful areas.

Finally, it is worth highlighting that the Lens is guided by standards to make data findable, accessible, interoperable, and reusable, the FAIR principles, which facilitates the maximal use of research data.

## CONCLUSION

Library support for an organization’s research can help improve patient care. Additionally, it can also help raise the profile and perceived value of the library service in the organization. Many health services have limited budgets for supporting research infrastructure and, thus, are unable to utilize many of the sophisticated research platforms that universities and research institutes use. In contrast, the Lens platform is free, and the process outlined here can be implemented at no cost. In addition to providing a platform for an organization’s research output, this approach also provides additional value such as research metrics and citation impact and, thus, could be a useful resource for health service libraries.

## 

***Rob Penfold****, rob.penfold@barwonhealth.org.au, https://orcid.org/0000-0002-5879-7388, Director, Library and Literacy, Barwon Health, Geelong, VIC, Australia*
